# Data Acquisition Control for UAV-Enabled Wireless Rechargeable Sensor Networks

**DOI:** 10.3390/s23073582

**Published:** 2023-03-29

**Authors:** Ikjune Yoon

**Affiliations:** Division of AI Computer Science and Engineering, Kyonggi University, Suwon-si 16227, Republic of Korea; ijyoon@kyonggi.ac.kr; Tel.: +82-31-249-9642

**Keywords:** wireless sensor network, mobile sink, sensing rate, wireless power transfer, unmanned aerial vehicle, energy aware, data acquisition control

## Abstract

In the realm of Internet of Things (IoT), wireless sensor networks (WSNs) have been the subject of ongoing research into the use of energy harvesting to capture ambient energy, and wireless power transfer (WPT) via a mobile charger to overcome the energy limitations of sensors. Moreover, to mitigate energy imbalance and reduce the number of hops, strategies have been developed to leverage cars or unmanned aerial vehicles (UAVs) as mobile sinks. The primary objective of this work is to increase network lifetime by reducing energy consumption of hotspot nodes and increasing the amount of data acquired from all sensors in an environment that combines the methods mentioned above.To achieve this objective, the proposed method involves developing multiple minimum depth trees (MDTs) for all nodes, considering the energy of the UAV and sensor nodes. Parent nodes prevent their own energy depletion and ensure data transmission without imbalance by adaptively controlling the data sensed at the nodes and their child nodes. Consequently, the energy depletion of nodes in hotspots is prevented, more sensory data is acquired, and balanced data collection from all nodes is achieved. Simulation results demonstrate that the proposed scheme outperforms other state-of-the-art methods in terms of reduced energy depletion, increased network connectivity, and the amount of data collected at the sink node. This scheme will be applied to applications that collect environmental data outdoors, such as climate measurement, to collect data uniformly and increase the lifespan of the network, thereby reducing network maintenance costs while collecting data effectively.

## 1. Introduction

In theInternet of Things (IoT), wireless sensor networks (WSNs) have been widely utilized to facilitate the collection of a large amount of data from areas that are difficult for humans to access or cover over a wide area. In WSNs, many small wireless sensor nodes are deployed for collection of environmental information; however, since the nodes run on batteries, the lifetime of the network is limited. To increase network lifetime, the batteries of the sensor nodes need to be replaced or recharged; however, due to the deployment of WSNs in areas that are difficult for humans to access or cover over a wide area, practical implementation of battery replacement or recharging is challenging. Therefore, there has been ongoing research aimed at developing methods to enhance energy efficiency and prolong the network lifetime of WSNs [1,2].

One potential solution to address the limited energy of sensor nodes is to leverage energy-harvesting techniques that capture and utilize energy obtained from the surrounding environment. In this method, energy is harvested from the ambient environment for the sensor nodes in the form of, for example, solar energy [3,4], wind energy [5,6], temperature difference [7], and vibration energy [8,9]. Among the different types of ambient energy, solar energy is a preferred source for sensor nodes because of its high energy density; however, the amount of harvested energy is highly variable depending on time, weather, and seasonal factors. To address this problem, techniques for prediction of available harvested energy considering factors such as weather, season, and time [10,11] or methods of allocating available based on time-slots [12,13] have been investigated or developed.

The application of energy harvesting allows to some extent the mitigation of the limited energy available for sensor nodes; however, with recent technological development, these sensors have been actively applied for acquisition of a large volume of data or multimedia-type data such as sound, photos, or videos, and transmission of such data requires high transmission efficiency and considerable energy consumption. Since the amount of energy obtained by energy-harvesting method is limited, research is currently underway on the utilization of wireless power transfer (WPT) as an additional means for supplying energy to the sensor nodes in such WSNs [14,15]. WPT can be implemented using inductive coupling, magnetic resonance coupling, and radio frequency (RF)-based WPT systems [14,16].

For application of WPT in WSNs, RF-based systems with a base station can be used for energy transfer over an extensive area [17,18] or mobile chargers based on UAVs can be used for physically visiting sensor nodes and recharging them [19,20]. The use of a base station allows recharging of a large number of sensor nodes at once; however, due to the poor efficiency of energy transfer and a rapid decrease in efficiency with distance, the network may end up with a severe energy imbalance. In contrast, WPT via a mobile charger allows for efficient energy transfer even for a large amount of energy. However, it is practically impossible for a mobile charger to visit all of sensor nodes in large numbers; moreover, the energy available for charging with a mobile charger is also limited. Recent studies are exploring techniques to overcoming these limitations.

Another problem in WSNs involves hotspots in which data are concentrated on a specific node [21,22]. Nodes in hotspots often experience fast energy depletion compared to other nodes due to the large amount of data transmission that occurs at the hotspot node, such as a sink node or the neighboring nodes where data is concentrated.

If blackout occurs in a node in the hotspot and the node ceases operation, the data of other nodes that were relayed by the node also cannot be sent to the sink node, which may result in the loss of a large amount of data. In addition, for nodes not located in a hotspot, even in the case of high energy availability of the nodes, these other nodes cannot have their data transmitted because there is no node available for relaying their data. This inevitably leads to the problem of energy imbalance, where surplus energy remains unused. Consequently, the problem of energy imbalance may lead to data imbalance in which sensing data of certain area in the network are actively collected whereas the data from other areas in the network are not collected.

A conventional approach for addressing the energy imbalance problem is a routing-based method [23,24]. This method aims to attain energy balance by optimizing the data transmission path to nodes with high energy availability. The drawback of the routing method is its limitations to addressing the problem of energy shortage in a sink node and its neighboring nodes. Another approach for addressing the energy imbalance issue is to use a mobile sink node [25,26]. In this method, a sink node located in a hotspot moves to different locations, thereby reducing the data transmission path and alleviating the energy imbalance problem. In addition, research is underway to develop various techniques aimed at resolving the problem of energy imbalance in the network.

This paper proposes a data acquisition control method for mitigating the energy imbalance problem in the network environment of UAV-enabled wireless rechargeable sensor networks (WRSNs) where energy is derived from ambient environment and by WPT. This method aims to achieve an increased network lifetime as well as an increase in the amount of data acquired. In the proposed scheme, a UAV equipped with a wireless charger is used as a mobile sink node that periodically traverses the network along a predetermined path, and some of the sensor nodes in this path are selected as the root nodes of the minimum depth trees (MDTs) to form MDTs. The sensor nodes send their own sensing data to the root node, and the UAV visits the root node to collect the data gathered at the root node while wirelessly charging the root node. In this process, the root node collects information on the number of its descendant nodes and the amount of data that can be transmitted to determine the amount of data to be sensed at the root node and the descendant nodes along with the period of such data collection. By allocating the determined amount of data, the proposed scheme prevents descendant nodes from transmitting data in excess of the allocated amount of data. In this way, energy depletion of the node in a hotspot is minimized, the problem of energy imbalance is mitigated, thereby enabling collection of a larger amount of data as well as balanced data collection across all nodes involved.

The main contributions of our paper are as follows:

- Most of the studies for WRSNs consider the case of harvesting energy from the surrounding environment or the mobile charger, but we address an environment in which sensor nodes harvest energy from both.
- Considering the energy obtained from energy harvesting and WPT, and the energy allocated by time, the algorithm that each node determines the amount of data it can sense and transmit is proposed. As a result, each node can collect uniform data over time without blackout.
- The parent nodes collect more information than other existing schemes, which is the amount of data that descendent nodes can transmit and the number of descendent nodes, and based on this, determines the amount of data sensed by all nodes more accurately.
- By limiting the amount of data transmission of descendant nodes, the burden of parent nodes to transmit is reduced, preventing nodes in the hotspot from blackout. As a result, the data sensed by each node is successfully delivered to the sink node and geographically uniform data is obtained.

In our previous work [27] based on the same environmental setting as this study, we performed clustering of nodes and determined the amount of data sensed at each node, thereby preventing energy depletion of root nodes and increasing the amount of data collected. However, in our previous work, the amount of data collection was predicted using the total number of nodes per hop distance, resulting in compromised accuracy in prediction. Additionally, the data sensed at each node was not transmitted after being gathered by relay nodes but only those cases of direct transmission to the root node were considered in the previous scheme, resulting in drawbacks in terms of throughput and energy efficiency. The proposed scheme addresses the limitations of the previous scheme, and the root node collects the number of descendant nodes and amount of data that can be transmitted. Based on this information, the amount of data that can be sensed at each child node is accurately calculated and broadcast to child nodes. Consequently, the energy depletion of parent nodes is effectively suppressed. In addition, throughput and energy efficiency are increased by considering the case where the data of descendant nodes is temporarily aggregated and transmitted by their parent nodes.

The remainder of this paper is organized as follows. In Section 2, we introduce the existing schemes related to WRSNs. Section 3 covers the details of the proposed scheme. In Section 4, we present the results of the performance evaluation of the proposed scheme, while Section 5 concludes the work.

## 2. Related Work

WPT is a technology that enables energy transfer over long distances, and recent trends demonstrate its active application in diverse fields such as various mobile devices, vehicles, and UAVs [16]. Research on WPT-enabled WSNs has also been actively carried out [28].

As mentioned earlier, one WPT method applied in WSNs is the use of a RF-based system for WPT from a base station. The method allows power transfer to many nodes located far from the base station; however, WPT efficiency is low and it decreases sharply with distance. Eidaks et al. [17] and Har [29] proposed multi-hop WPT to increase the efficiency of RF-based WPT by implementing intensive energy transfer to the hotspot. Specifically, Eidaks et al. [17] investigated the possibility of multi-hop WPT through experiments by constructing hardware capable of using multi-hop WPT. These two schemes have the pros of being able to charge a large number of sensor nodes at once with higher energy efficiency than general RF-based WPT. Nevertheless, the energy efficiency is still low and the sensor node consumes a lot of energy to relay the data of other sensor nodes. In our proposed method, the sink node collects data from the sensor node and transfers energy at the same time, and therefore, Therefore, the energy transfer efficiency is higher and the sensor node consumes less energy to relay data than these two schemes.

Perera et al. [18] and Ejaz et al. [30] proposed a method for energy transfer to sensor nodes by setting up charging base stations in several places in the network for implementation of RF WPT. These two methods presented a method of deploying base stations to achieve efficient WPT to all nodes. These schemes using base stations mentioned above have the disadvantages of low WPT efficiency and difficulty in actively coping with energy imbalance between sensor nodes or hotspot problem. To improve this shortcoming, we approached the imbalance problem more flexibly by using a mobile charger.

Unlike the above methods with the base station, for local and intensive energy transfer to specific nodes, research has been undertaken on methods of installing a wireless charger in a car or UAV and physically visiting nodes for charging. This approach allows improved energy efficiency by implementing WPT in a short distance through inductive coupling or magnetic resonance coupling as well as RF. This type of mobile chargers not only perform WPT by visiting nodes but also serve as mobile sinks for collecting data, thereby helping address the energy imbalance problem. However, the energy capacity of these mobile chargers is also limited, and they consume energy while moving or traveling, leading to limitations in terms of the number of nodes that can be visited.

Mobile chargers are generally categorized into two types, car based and UAV based. While using a car has the advantage in terms of carrying more energy than a UAV, it has limitations in terms of mobility and access to certain areas that are difficult to reach. Using a UAV, on the other hand, allows quick movement and better reach in inaccessible areas, but the flight requires considerable energy consumption and a UAV cannot carry as much energy as a car because of its payload limitations.

Guo et al. [19] and Tu et al. [20] proposed methods that used a car as a mobile charger. When a car travels a set route and charges nodes, their proposed methods aim to increase the network lifetime by increasing the efficiency of WPT. Of these studies, the method proposed by Tu et al. [20] determines the charging method by considering the energy demand and charging time of the sensor nodes. These two methods have the disadvantages that they are vulnerable to terrain because they use vehicles as mobile chargers, and they cannot be applied to an environment with many nodes because the car has to traverse all nodes with a limited time and energy. Sangare et al. [31] proposed a method for setting the number of nodes, the distance between nodes, and the distance between the nodes and a charger to facilitate efficient WPT in a system for transferring energy to wireless sensor nodes using the RF method. In addition, for validation of the proposed method, a prototype using a car was developed. In this method, only a small number of sensor nodes are deployed at predetermined locations, and a car traverses them and transfers energy. This method, like the above two methods, cannot be applied to an environment with many sensor nodes and is vulnerable to terrain.

Next, we describe the use of UAV-based mobile wireless chargers. Xu et al. [32] and Baek et al. [33] proposed methods aimed at optimizing the path and hovering time of a UAV in an environment that involves simultaneous data collection with UAV and RF-based WPT to sensor nodes. The methods consider the energy consumption of sensor nodes and harvested energy within the energy limit of a UAV, with the aim of maximizing the energy of the sensor nodes. However, these methods have the disadvantage that individual sensor nodes can recevie only a small amount energy because UAV cannot hover for a long time with limited energy and the distance between sensor nodes and the UAV is long. To improve this disadvantage, we increased energy transfer efficiency by intensively delivering energy to a few sensor nodes and collecting energy from the surrounding environment for other nodes. Hu et al. [34] proposed a method of traversing all sensor nodes with a UAV, recharging them, and thereby preventing all nodes from blackout. This technique aims to minimize the energy consumed by the UAV and prevents all nodes from blackout using only the initial remaining network lifetime information, and determines the charging schedule and charging amount accordingly. This method has the advantage that it does not require much information to determine the charging schedule, but has the disadvantage that the prediction error may increase over time. Our scheme uses a method to more accurately measure the state of the WSN by collecting a little more information of each sensor node. In Chen et al. [35], unlike the cases in the above methods, when the UAV cannot traverse the entire network at once due to its energy limits, a method of installing a charging pad inside the network so that the UAV can be recharged in the middle of the process was presented. Additionally, the method involves determining the location and number of pads to increase energy efficiency.

Most of the above techniques are designed for environments using WPT alone, but some methods have been proposed for WSNs based on both WPT and energy-harvesting techniques. La Rosa et al. [36] proposed the hardware design of sensor nodes that leverages energy harvesting and WPT. They also proposed a method of supplying energy to a battery-free device and reported on the feasibility of the proposed methods. Jadhav and Lambor [37] proposed a hardware structure integrating an antenna capable of receiving RF-based WPT and a solar energy-harvesting function, and demonstrated the feasibility of the proposed methods. In Wang et al. [38], a hybrid framework with three-level topology that combines the use of wireless charging and solar energy harvesting was presented in a network composed of energy-harvesting nodes and battery-based nodes. In this method, since the high energy demand of the root node may not be met through power transfer, solar energy harvesting was employed for stable network operation. This scheme has the disadvantage that it cannot prevent blackout of neighboring nodes around the root node by using the solar energy collection node only for the root node. Conversely, our scheme uses a strategy in which all sensor nodes harvest energy from the environment and the hotspot nodes are charged by the mobile charger. This strategy can be applied to WSNs that need to transmit a large amount of sensing data by increasing the available energy of all sensor nodes.

Tsoumanis et al. [39] proposed an energy recharging policy based on local information only to solve the energy and distance median problems that optimize the consumed energy and the distance covered by a mobile recharger in rechargeable wireless sensor networks. Liu et al. [40] proposed a far-relay approach for WSN using mobile sink node with mobile charger which moves a pre-defined path in order to solve the joint data gathering and energy harvesting problem. In this approach, the sensor nodes closer to the path may allocate some of the harvested energy to assist the data transmission of other sensor nodes that are farther away. Under the far-relay approach, they also proposed an optimal scheduling scheme to maximize network utility. Liu et al. [41] determined the optimal number of sensor nodes to deploy to ensure a given coverage quality to monitor given target locations. They formulate the problem as an integer linear programming and use it to compute the minimum number of sensor nodes required to monitor targets. Then, they relax the integer variables of the integer linear programming and devise three approximation algorithms for large-scale WSNs. These schemes focus only on the method of charging node energy using WPT and energy harvesting to prevent blackout of energy depletion. Unlike these schemes, our proposed scheme aims to obtain data efficiently by using the harvested energy to the maximum. In particular, energy exhaustion of nodes in the hotspot is prevented so that data of nodes further away from the root node is not lost and geographically uniform data is obtained.

## 3. Data Acquisition Control Scheme

**Figure 1:**
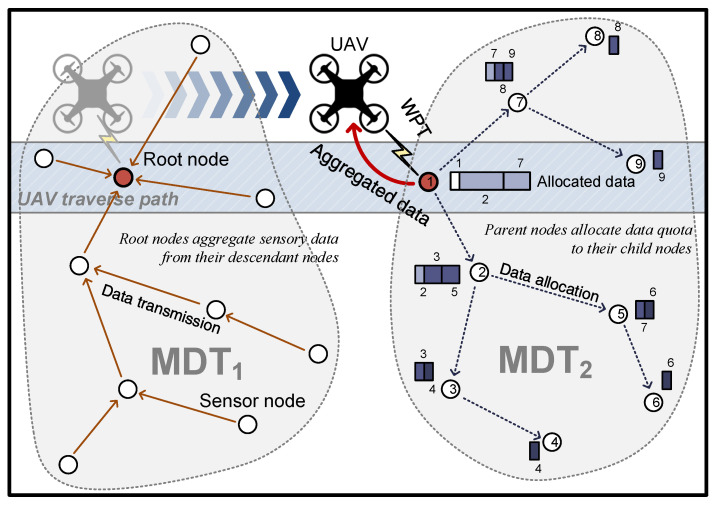
Overview of the proposed scheme.

This work focuses on WRSNs composed of energy-harvesting sensor nodes that derive energy from the ambient environment. The proposed method considers an environmental setting where a UAV visits the nodes to collect data and recharge the energy of the sensor nodes via WPT. The amount of data to be collected is determined based on the predicted available energy and energy consumption to mitigate the energy imbalance problem and maximize network connectivity and the amount of data collected. The UAV used in the proposed scheme traverses the predetermined path as in Shin et al. [42] and acts as a mobile sink node that gathers data from sensor nodes; at the same time, it is equipped with a wireless charger and transfers energy to sensor nodes using WPT. With limited energy, the UAV is unable to visit all nodes for gathering data; thus, the nodes along the path of the UAV are selected as root nodes of MDTs so that the UAV can visit only these root nodes for gathering data, and they form MDTs. In addition, since root nodes consume more energy than other nodes, energy is transferred to these nodes through WPT for recharging. The root node collects the number of descendant nodes and amount of data that can be transmitted. Based on this information, the amount of data that can be sensed at each child node is calculated and broadcast to child nodes. When all nodes from the root node to the leaf node are allocated with the information of the amount of data that can be transmitted, each node performs sensing of data less than the allocated amount and sends the data accordingly. In this manner, energy depletion of parent nodes is prevented and collection of the maximum amount of data is attained. Figure 1 gives an outline of the proposed scheme.

### 3.1. Energy Models

In the proposed scheme, the amount of data that can be collected is determined according to the predicted available energy at a sensor node and the number of descendant nodes dependent on the sensor node for data transmission. The sensor nodes consume energy primarily for data transmission and are charged through energy harvesting or by WPT. In the proposed scheme, a UAV with limited energy traverses the network and wirelessly recharges all nodes. Therefore, the available energy of the sensor nodes and the UAV needs to be predicted. To this end, in this subsection, we introduce the energy models of the sensor nodes and UAV used in the proposed scheme.

#### 3.1.1. Energy Model of the Sensor Nodes

The wireless sensor nodes, the main subjects of the proposed scheme, harvest solar energy, store it in a battery, and then use the stored energy for their operations. The solar energy is highly variable with time due to its nature, and therefore, to utilize the harvested energy without restriction of time, solar energy prediction methods as proposed in pro-energy [11] are employed in which estimation of future energy availability is estimated over time based on the past energy observations. Alternatively, an energy allocation scheme [12] is employed in which available energy is allocated based on time-slots. In the proposed scheme, periodic clustering and routing for sensor nodes are implemented, setting one round as the period, and available energy is allocated for each round. Since we aim for continuous operation of sensor nodes without energy depletion, the energy consumed at the sensor node per round (${e_{}}_{c}^{}$) must be less than or equal to the energy available per round (${e_{}}_{\mathrm{avail}}^{}$). That is, the following relationship of inequality must be satisfied to achieve continuous operation of sensor nodes without energy depletion:

(1) $${e_{}}_{\mathrm{avail}}^{}\geq {e_{}}_{c}^{},$$

where ${e_{}}_{\mathrm{avail}}^{}$ represents the energy allocated by the above-described energy allocation scheme considering the harvested solar energy and also the energy transferred by WPT.

Sensor nodes consume considerable energy for data transmission and reception as well as other operations for operations in circuits. Therefore, the energy consumption ${e_{}}_{c}^{}$ consists of the energy for data transmission ${e_{}}_{\mathrm{Tx}}^{}$, data reception ${e_{}}_{\mathrm{Rx}}^{}$, and for other operations requiring energy consumption ${e_{}}_{\mathrm{idle}}^{}$. Substituting these components into Equation (1), we have:

(2) $${e_{}}_{\mathrm{avail}}^{}\geq {e_{}}_{\mathrm{Tx}}^{}+{e_{}}_{\mathrm{Rx}}^{}+{e_{}}_{\mathrm{idle}}^{}.$$

Here, ${e_{}}_{\mathrm{Tx}}^{}$ can also be expressed as follows [43]:

(3) $${e_{}}_{\mathrm{Tx}}^{}=s_{}\beta {r_{}}^{\alpha },$$

where $s_{}$ denotes the size of data for transmission (bits), β is the energy consumption for transmission per bit according to the distance ($J/\mathrm{bit}/m^{\alpha }$), $r_{}$ is the transmission distance, and α indicates path loss (2–5). Since individual communication protocols have a limit on the maximum payload for one packet, if a sensor node has aggregated data of size ${s_{}}_{\mathrm{aggr}}^{}$, the data needs to be divided into multiple packets for transmission. If the maximum payload size of a packet is ${s_{}}_{\max}^{}$, the sensor data needs to be divided into data of size ${s_{}}_{\max}^{}$, and overheads such as header or trailer should be added to each packet before transmission. That is, for transmission of the sensor data of the size ${s_{}}_{\mathrm{aggr}}^{}$, the data packet size ${s_{}}_{\mathrm{packet}}^{}$ for actual transmission at the node is as follows:

(4) $${s_{}}_{\mathrm{packet}}^{}=\left\lceil \frac{{s_{}}_{\mathrm{aggr}}^{}}{{s_{}}_{\max}^{}}\right\rceil s_{\mathrm{head}}+{s_{}}_{\mathrm{aggr}}^{},$$

where $s_{\mathrm{head}}$ denotes the size of the header and trailer, excluding the payload, in the packet. Therefore, by substituting the packet of Equation (4) into Equation (3), we obtain the energy ${e_{}}_{\mathrm{Tx}}^{}$ required for transmission of data, ${s_{}}_{\mathrm{aggr}}^{}$, as follows:

(5) $${e_{}}_{\mathrm{Tx}}^{}=\left(\left\lceil \frac{{s_{}}_{\mathrm{aggr}}^{}}{{s_{}}_{\max}^{}}\right\rceil s_{\mathrm{head}}+{s_{}}_{\mathrm{aggr}}^{}\right)\beta {r_{}}^{\alpha }.$$

#### 3.1.2. Energy Model of the UAV

The UAV travels along a set path, visits a root node to gather data, and charges them via WPT. The UAV consumes energy for flight and data collection, and the remaining energy can be used for WPT. Therefore, to calculate the energy available for WPT, the energy consumed for movement of UAV and data collection needs to be considered first. If the UAV visits $m_{}$ number of root nodes for data gathering and energy transfer, to ensure safe traversing of the network without depleting the energy of the UAV, the following equation of inequality needs to be satisfied [44]:

(6) $${e_{}}_{\mathrm{full}}^{}-{e_{}}_{\mathrm{move}}-m_{}\left({e_{}}_{\mathrm{land}}^{}+{e_{}}_{\mathrm{Rx}}^{}\right)-{e_{}}_{\mathrm{uidle}}\geq 0,$$

where ${e_{}}_{\mathrm{full}}^{}$ denotes the total energy of the UAV, move is the energy consumed in flight ${e_{}}_{\mathrm{land}}^{}$ is the energy consumed in landing/takeoff, ${e_{}}_{\mathrm{Rx}}^{}$ is the energy required for data reception, and ${e_{}}_{\mathrm{uidle}}$ represents the energy consumed in other electronic equipment. However, the UAV transfers the surplus energy to the sensor node by WPT. That is, the energy ${e_{}}_{\mathrm{charge}}$ that can be used for WPT can be expressed as follows:

(7) $${e_{}}_{\mathrm{charge}}\leq {e_{}}_{\mathrm{full}}^{}-{e_{}}_{\mathrm{move}}-m_{}\left({e_{}}_{\mathrm{land}}^{}+{e_{}}_{\mathrm{Rx}}^{}\right)-{e_{}}_{\mathrm{uidle}}.$$

In the above equation, values of ${e_{}}_{\mathrm{full}}^{}$, ${e_{}}_{\mathrm{uidle}}$ and ${e_{}}_{\mathrm{Rx}}^{}$ depend on the specifications of the UAV, and ${e_{}}_{\mathrm{move}}$ and ${e_{}}_{\mathrm{land}}^{}$ are determined according to the network environment. Thus, based on the above equation, in our target environment, ${e_{}}_{\mathrm{charge}}$ can be determined by $m_{}$.

### 3.2. Determining the Amount of Data Sensed at Each Sensor Node

**Figure 2:**
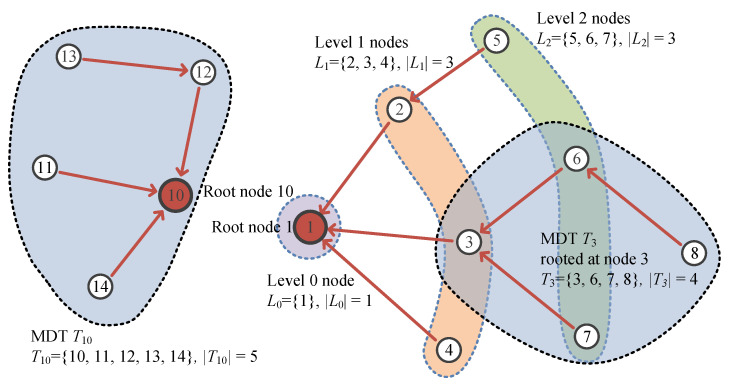
Example of MDT with presentation of key variables.

In this subsection, we describe how each sensor node determines the amount of data sensed at the respective node. In this subsection, $T_{i}$ represents a set of nodes in a subtree rooted at node *i* and $|T_{i}|$ is the number of members of $T_{i}$. Figure 2 presents an example of MDT using the equations used in this paper.

#### 3.2.1. The Amount of Data Sensed

The proposed scheme aims to achieve balanced data collection for all nodes within the limited energy available. Therefore, if the amount of data sensed at each node per round is represented as ${s_{}}_{\mathrm{sense}}^{}$, then the amount of data that needs to be transmitted per round for node *i*, ${{s_{}}_{\mathrm{aggr}}^{}}_{i}$, can be expressed as follows:

(8) $${{s_{}}_{\mathrm{aggr}}^{}}_{i}\approx |T_{i}|{s_{}}_{\mathrm{sense}}^{}.$$

In the previous study, we calculated ${s_{}}_{\mathrm{sense}}^{}$, the amount of data that a sensor node with level $l_{}$, which is $l_{}$ hops away from the root node, can transmit per round as follows:

(9) $${s_{}}_{\mathrm{sense}}^{}\leq \frac{{e_{}}_{\mathrm{avail}}^{}-{e_{}}_{\mathrm{Rx}}^{}-{e_{}}_{\mathrm{idle}}^{}}{|T_{i}|\beta {r_{}}^{\alpha }},$$

where $L_{}$ is a set of all nodes in level $l_{}$ in the MDT, $|L_{l_{}}|$ is the number of all nodes of $L_{}$, and $l_{\max}$ denotes the maximum level. In this scheme of the previous study, the amount of data transmission refers to the case when the data are not aggregated but each data is separately transmitted to the root node. The problem with this method is that the throughput is lowered due to the addition of header and trailer of the packet for each data. In this study, for improved performance, nodes located along the path to the root node receive data from their descendant nodes and then aggregate the data and transmit them to the parent node. Therefore, Equation (9) needs to be modified accordingly.

First, the following equation can be derived using Equations (2) and (5):

(10) $${e_{}}_{\mathrm{avail}}^{}\geq \left(\left\lceil \frac{{s_{}}_{\mathrm{aggr}}^{}}{{s_{}}_{\max}^{}}\right\rceil s_{\mathrm{head}}+{s_{}}_{\mathrm{aggr}}^{}\right)\beta {r_{}}^{\alpha }+{e_{}}_{\mathrm{Rx}}^{}+{e_{}}_{\mathrm{idle}}^{},$$

where $\left\lceil \frac{{s_{}}_{\mathrm{aggr}}^{}}{{s_{}}_{\max}^{}}\right\rceil$ denotes the actual number of data packets for transmission, and this can be expressed as $\frac{{s_{}}_{\mathrm{aggr}}^{}}{{s_{}}_{\max}^{}}+1$ in many cases. Thus, Equation (10) can be reorganized and represented as follows:

(11) $${e_{}}_{\mathrm{avail}}^{}\geq \left(\left(\frac{{s_{}}_{\mathrm{aggr}}^{}}{{s_{}}_{\max}^{}}+1\right)s_{\mathrm{head}}+{s_{}}_{\mathrm{aggr}}^{}\right)\beta {r_{}}^{\alpha }+{e_{}}_{\mathrm{Rx}}^{}+{e_{}}_{\mathrm{idle}}^{}.$$

Rewriting the above equation for ${s_{}}_{\mathrm{aggr}}^{}$ gives the following:

(12) $${s_{}}_{\mathrm{aggr}}^{}\leq \frac{\left({e_{}}_{\mathrm{avail}}^{}-{e_{}}_{\mathrm{Rx}}^{}-{e_{}}_{\mathrm{idle}}^{}\right)/\left(\beta {r_{}}^{\alpha }\right)-s_{\mathrm{head}}}{s_{\mathrm{head}}/{s_{}}_{\max}^{}+1}.$$

Therefore, to enable transmission of maximum number of data under the condition that the corresponding sensor node does not go blackout, ${s_{}}_{\mathrm{aggr}}^{}$ must be represented as the right side of Equation (12).

Energy that can be used per round is allocated based on the energy allocation scheme according to the time for a sensor node, and the node must use the energy equal to or less than the amount of allocated energy. That is, ${e_{}}_{\mathrm{avail}}^{}$ equals to the energy allocated for that round, ${e_{}}_{\mathrm{alloc}}$. On the contrary, in the case of root nodes, unlike other sensor nodes, energy that is transferred with WPT by the UAV can be used in addition to the energy allocated. If the number of MDTs is $m_{}$ and the UAV equally distributes the remaining energy to all root nodes as ${e_{}}_{\mathrm{charge}}$, the amount of energy received by one root node is $\eta {e_{}}_{\mathrm{charge}}/m_{}$, and the total available energy for one root node is expressed as follows:

(13) $${e_{}}_{\mathrm{alloc}}+\eta \frac{{e_{}}_{\mathrm{charge}}}{m_{}},$$

where η is the WPT efficiency. Therefore, ${e_{}}_{\mathrm{avail}}^{}$, the energy that a sensor node of level $l_{}$ can use during one round can be expressed as follows:

(14) $${e_{}}_{\mathrm{avail}}^{}=\left\{\begin{array}{cc} {e_{}}_{\mathrm{alloc}}, & \mathrm{if}\;\mathrm{the}\;\mathrm{node}\;\mathrm{is}\;a\;\mathrm{root}\;\mathrm{node}\\ {e_{}}_{\mathrm{alloc}}+\eta \frac{{e_{}}_{\mathrm{charge}}}{m_{}}, & \mathrm{otherwise}. \end{array}\right.$$

By substituting ${e_{}}_{\mathrm{avail}}^{}$ into Equation (12), ${s_{}}_{\mathrm{aggr}}^{}$, the amount of data that can be sensed per round at the sensor node within the available energy, can be obtained.

${s_{}}_{\mathrm{aggr}}^{}$ is the amount of data aggregation for the node and its descendant nodes. For balanced data collection for the entire network, a limited amount of data needs to be allocated for shared use with the descendant nodes so that the same amount of sensing data is collected at the node and its descendant nodes. Therefore, the amount of sensing data by a single node *i* per round (${{s_{}}_{\mathrm{sense}}^{}}_{i}$) can be expressed as:

(15) $${{s_{}}_{\mathrm{sense}}^{}}_{i}=\frac{{{s_{}}_{\mathrm{aggr}}^{}}_{i}}{|T_{i}|}.$$

Therefore, ${{s_{}}_{\mathrm{sense}}^{}}_{i}$ represents the amount of data that can be sensed at node *i* and its descendant nodes, respectively.

#### 3.2.2. Determining the Number of MDTs

As discussed previously, to enable efficient data collection, estimation of available energy is derived for each node, and the amount of sensing data within the range of available energy needs to be determined. To this end, $m_{}$ must be calculated first to determine ${e_{}}_{\mathrm{charge}}$ of Equation (7) and ${s_{}}_{\mathrm{aggr}}^{}$ of Equation (12).

The method of deriving $m_{}$ is based on a method similar to the method proposed in our previous study [27]. First, the range of $m_{}$ is limited by the condition that the energy of the UAV should not be exhausted, and the number of nodes per level in the network formed by $m_{}$ MDTs is predicted to obtain the expected number of descendant nodes of each node. Based on this information, the expected amount of sensor data ${\hat{s_{}}}_{\mathrm{sense}}$ that can be sensed at one node is calculated and the value of $m_{}$ that maximizes the amount of data is selected.

First, as for the range of $m_{}$ that allows operation of the UAV, $m_{}$ must be at least one, and the value of $m_{}$ must be determined within a range that does not exceed the available energy of the UAV. Considering the energy availability of the UAV in Equation (6), $m_{}$ should be determined within the following range:

(16) $$1\leq m_{}\leq \left\lfloor \frac{{e_{}}_{\mathrm{full}}^{}-{e_{}}_{\mathrm{move}}-{e_{}}_{\mathrm{idle}}^{}}{{e_{}}_{\mathrm{land}}^{}+{e_{}}_{\mathrm{Rx}}^{}}\right\rfloor .$$

Second, $m_{}$ that maximizes the amount of data sensed at the sensor node needs to be derived. Since the number of nodes constituting one MDT and the maximum hop distance vary according to the number of MDTs, the amount of data to be transmitted differs even though the sensor nodes sensed the same amount of data. Therefore, $m_{}$ needs to be determined as the value that maximizes the number of data sensed at the nodes.

We now calculate the number of nodes for each level to derive the number of descendant nodes for data transmission from the parent node. Son et al. [45] derived the expected number of nodes $|\hat{L_{l_{}}}|$ at level $l_{}$ of a single MDT when nodes are evenly distributed as follows:

(17) $$|\hat{L_{l_{}}}|=\left\{\begin{array}{cc} 1 & \mathrm{if}\;l_{}=0\\ \min\left(\frac{n^{}}{m_{}},\rho \pi {l_{}}^{2}{r_{}}^{2}\right)-\sum_{j=0}^{l_{}-1}|\hat{L_{j}}| & \mathrm{if}\;l_{}>0, \end{array}\right.$$

where $n^{}$ denotes the total number of nodes, ρ is the node density ($n^{}/m^{2}$), and $r_{}$ indicates the transmission distance of the node. The $|\hat{L_{l_{}}}|$ nodes at the distance of $l_{}$ are responsible for transmission of their own data as well as those of $\sum_{j=l_{}+1}^{l_{\max}}|\hat{L_{j}}|$ descendant nodes. That is, a single node at level $l_{}$ is responsible for transmission of $\sum_{j=l_{}+1}^{l_{\max}}|\hat{L_{j}}|/|\hat{L_{l}}|$ descendant nodes on average; thus, the predicted number of nodes $|\hat{T_{i}}|$ for which a node *i* at level $l_{}$ is responsible for data transmission is expressed as follows:

(18) $$|\hat{T_{i}}|=\frac{\sum_{j=l_{}+1}^{l_{\max}}|\hat{L_{j}}|}{|\hat{L_{l}}|}+1.$$

Meanwhile, the nodes that become the bottle neck in WSNs are the root node and level one nodes [46]. We need to determine the maximum amount of data that the root node and level one nodes can transmit during one round to determine the MDT that maximizes data collection. First, by substituting ${s_{}}_{\mathrm{aggr}}^{}$ of Equation (12) into (19), ${\hat{s_{}}}_{\mathrm{sense}}$, the predicted amount of sensor data that can be collected per node can be expressed as follows:

(19) $${\hat{s_{}}}_{\mathrm{sense}}\leq \frac{\left({e_{}}_{\mathrm{avail}}^{}-{e_{}}_{\mathrm{Rx}}^{}-{e_{}}_{\mathrm{idle}}^{}\right)/\left(\beta {r_{}}^{\alpha }\right)-s_{\mathrm{head}}}{\left(s_{\mathrm{head}}/{s_{}}_{\max}^{}+1\right)|\hat{T_{l}}|}.$$

By substituting Equation (14) into Equation (19), ${\hat{s_{}}}_{\mathrm{sense}}{}_{i}$ can be expressed as follows by dividing the cases into when node *i* is the root node and when it is a node at level one.

(20) $${\hat{s_{}}}_{\mathrm{sense}}{}_{i}\leq \left\{\begin{array}{cc} \frac{\left({e_{}}_{\mathrm{alloc}}+\eta {e_{}}_{\mathrm{charge}}/m_{}-{e_{}}_{\mathrm{Rx}}^{}-{e_{}}_{\mathrm{idle}}^{}\right)/\left(\beta {r_{}}^{\alpha }\right)-s_{\mathrm{head}}}{\left(s_{\mathrm{head}}/{s_{}}_{\max}^{}+1\right)|\hat{T_{0}}|}, & \mathrm{if}\;i\in T_{0}\\ \frac{\left({e_{}}_{\mathrm{alloc}}-{e_{}}_{\mathrm{Rx}}^{}-{e_{}}_{\mathrm{idle}}^{}\right)/\left(\beta {r_{}}^{\alpha }\right)-s_{\mathrm{head}}}{\left(s_{\mathrm{head}}/{s_{}}_{\max}^{}+1\right)|\hat{T_{1}}|}, & \mathrm{if}\;i\in T_{1}. \end{array}\right.$$

We determine $m_{}$ that maximizes ${\hat{s_{}}}_{\mathrm{sense}}$ of Equation (16) as the number of MDTs in the round, within the range of $m_{}$ in Equation (20) given above.

Once the number of MDTs is determined, the root node of each MDT needs to be selected. In order to reduce the number of transmission hops of all nodes, the root node needs to be evenly distributed. Therefore, in the proposed scheme, the root node is selected among nodes deployed at regular intervals on the path of the UAV. In addition, by selecting a root node at a random location, the energy balance of all nodes is increased. By applying the head node selection process proposed in our previous study, we first select a random location in the path of the UAV and then set the root node candidate area at equal intervals from the selected location. Then, the node with the largest ${\hat{s_{}}}_{\mathrm{sense}}$ is determined as the root node among the nodes in the set area.

#### 3.2.3. Creating MDTs and Routing

Once the number of MDTs and the root node of each MDT are determined, each root node creates their MDT for routing, the root node broadcasts a control message including its own ID and level to neighboring nodes by flooding, and the nodes that receive the message set the node that sent this message as its parent node and update their ID and level into the ID and level of the message received; subsequently, this message is again broadcast to the neighboring nodes. If a routing message is received again while a routing message has already been received, the node with the lower level is determined as the parent node after comparing with the level of the previous message. When the level is updated, this information is broadcast again so that other nodes are informed of the updated level to select a shorter path.

#### 3.2.4. Allocating the Amount of Data Sensed

After the process of routing, an accurate amount of data sensed at nodes needs to be calculated by considering the available energy. To collect as much data as possible within the range of not causing depletion in the energy of the parent node, the proposed scheme collects information from all nodes to the root node, and the amount of data sensed at each node is calculated for all nodes from the root node to the leaf nodes and this information is broadcast.

##### Collecting Information of All Nodes to Their Root Node

To calculate the accurate amount of data for transmission, sensor node *j* needs to send the amount of data ${{s_{}}_{\mathrm{aggr}}^{}}_{j}$, it can transmit during one round and $|T_{j}|$, the number of nodes it is responsible for data transmission to the parent node. Based on this information, the parent node *i* calculates its own ${{s_{}}_{\mathrm{aggr}}^{}}_{i}$ and $|T_{i}|$ and delivers this information to its parent node. When this process is iterated up to the root node, the root node calculates accurate ${s_{}}_{\mathrm{aggr}}^{}$ and broadcasts this information to the descendant nodes so that the amount of data to be sensed and the period can be determined for all descendant nodes.

The ${{s_{}}_{\mathrm{aggr}}^{}}_{j}$ to be delivered to the parent node is calculated using Equation (12), and $|T_{i}|$ can be obtained as follows:

(21) $$\begin{array}{cc} |T_{i}| & =\left\{\begin{array}{cc} 1 & \mathrm{if}\;\mathrm{node}\;i\;\mathrm{is}\;a\;\mathrm{leaf}\;\mathrm{node}\\ \sum_{j\in C_{i}}|T_{j}|+1 & \mathrm{otherwise} \end{array}\right. \end{array}$$

(22) $$\begin{array}{cc} C_{i} & =\{x|x\;\mathrm{is}\;\mathrm{the}\;\mathrm{ID}\;\mathrm{of}\;a\;\mathrm{child}\;\mathrm{node}\;\mathrm{of}\;\mathrm{node}\;i.\} \end{array}$$

Node *j* transmits ${{s_{}}_{\mathrm{aggr}}^{}}_{j}$ and $|T_{j}|$ calculated as above to the parent node. When the parent node *i* receives information from node *j*, it adds *j*, ${{s_{}}_{\mathrm{aggr}}^{}}_{j}$ and $|T_{j}|$ to $C_{i}$, ${S_{\mathrm{aggr}}^{}}_{i}$, and ${N_{\mathrm{sub}}^{}}_{i}$, respectively. Here, ${S_{\mathrm{aggr}}^{}}_{i}$ and ${N_{\mathrm{sub}}^{}}_{i}$ are the sets of ${s_{}}_{\mathrm{aggr}}^{}$ and $|T_{}|$ of node *i*’s children nodes, respectively. After receiving the information from all child nodes according to the above process, the node’s own ${{s_{}}_{\mathrm{aggr}}^{}}_{i}$ and $|T_{i}|$ are calculated and this information is sent to the parent node. Algorithm 1 shows the process of collecting information of all nodes to the root node as above.
**Algorithm 1:** Collecting information of all nodes to their root node.**Initialize:** ${C^{}}_{i}\leftarrow \emptyset$, ${S_{\mathrm{aggr}}^{}}_{i}$, $|T_{i}|\leftarrow 1$1:**repeat**2:    Node *i* receives ${{s_{}}_{\mathrm{aggr}}^{}}_{j}$ and $|T_{j}|$ from its child node *j*.3:    $|T_{i}|\leftarrow |T_{i}|+|T_{j}|$4:    ${C^{}}_{i}\leftarrow {C^{}}_{i}\cup \left\{j\right\}$5:    ${S_{\mathrm{aggr}}^{}}_{i}\leftarrow {S_{\mathrm{aggr}}^{}}_{i}\cup \left\{{{s_{}}_{\mathrm{aggr}}^{}}_{j}\right\}$6:    ${N_{\mathrm{sub}}^{}}_{i}\leftarrow {N_{\mathrm{sub}}^{}}_{i}\cup \left\{|T_{j}|\right\}$7:**until** Receiving information from all child nodes.8:**if** Node *i* is not a root node **then**9:      Node *i* sends ${{s_{}}_{\mathrm{aggr}}^{}}_{i}$ and $|T_{i}|$ to its parent node.10:**else**11:    The root node calculate its children’s ${s_{}}_{\mathrm{alloc}}^{}$ using Algorithm 2.12:**end if**

##### Allocating the Amount of Sensing Data for Collection

When the information regarding all nodes of MDT is received by the root node, the root node determines the amount of data that can be sensed from itself and child nodes and broadcasts this information to the child nodes. That is, node *i* needs to calculate ${S_{\mathrm{alloc}}^{}}_{i}$, which is a set consisting of ${{s_{}}_{\mathrm{alloc}}^{}}_{j}$, the amount of data that can be sensed by itself in this round and ${{s_{}}_{\mathrm{alloc}}^{}}_{j}$, the amount of data that can be sensed in this round by the child node *j* ($j\in {C_{}}_{i}$) of node *i*. The calculated ${S_{\mathrm{alloc}}^{}}_{i}$ needs to be broadcast to the child nodes.

First, node *i* determines ${{s_{}}_{\mathrm{alloc}}^{}}_{i}$ as ${{s_{}}_{\mathrm{aggr}}^{}}_{i}$. To calculate ${{s_{}}_{\mathrm{alloc}}^{}}_{j}$ of its child nodes, node *i* first calculates ${{s_{}}_{\mathrm{sense}}^{}}_{j}$ by substituting $|T_{i}|$ and the elements of ${S_{\mathrm{aggr}}^{}}_{i}$ into Equation (19) and then constructs ${S_{\mathrm{sense}}^{}}_{i}$ using the calculated ${{s_{}}_{\mathrm{sense}}^{}}_{j}$.

Case 1. If ${{s_{}}_{\mathrm{sense}}^{}}_{j}>{{s_{}}_{\mathrm{sense}}^{}}_{i}$, node *i* itself may be blackout, so ${{s_{}}_{\mathrm{sense}}^{}}_{j}$ needs to be limited to be equal to or less than ${{s_{}}_{\mathrm{sense}}^{}}_{i}$. That is, in order for $|T_{j}|$ nodes to transmit only the data of respective ${{s_{}}_{\mathrm{sense}}^{}}_{i}$, the ${{s_{}}_{\mathrm{alloc}}^{}}_{j}$ of node *j* is expressed as follows:

(23) $${{s_{}}_{\mathrm{alloc}}^{}}_{j}={{s_{}}_{\mathrm{sense}}^{}}_{i}|T_{j}|.$$

Since node *i* allocated data of size ${{s_{}}_{\mathrm{alloc}}^{}}_{j}$ to node *j* and its descendant nodes, this is excluded from ${{s_{}}_{\mathrm{alloc}}^{}}_{i}$ and $|T_{i}|$, respectively, and ${{s_{}}_{\mathrm{sense}}^{}}_{i}$ is recalculated.

Case 2. In the opposite case, if ${{s_{}}_{\mathrm{sense}}^{}}_{j}\leq {{s_{}}_{\mathrm{sense}}^{}}_{i}$, since the availability of the parent node is greater than the amount of data to be sent by node *j*, node *j* can send ${{s_{}}_{\mathrm{sense}}^{}}_{j}$ without making any change. In addition, since node *i* may transmit less data of *j* than predicted amount, the amount of remaining data can be allocated to other nodes. Therefore, the remaining data availability of node *i* is evenly allocated to other nodes. That is, ${{s_{}}_{\mathrm{alloc}}^{}}_{j}$ and $|T_{j}|$ of all *j*s where ${{s_{}}_{\mathrm{sense}}^{}}_{j}>{{s_{}}_{\mathrm{sense}}^{}}_{i}$ are excluded from ${{s_{}}_{\mathrm{alloc}}^{}}_{i}$ and $|T_{i}|$, respectively, then ${{s_{}}_{\mathrm{alloc}}^{}}_{i}$ is recalculated as follows:

(24) $${{s_{}}_{\mathrm{alloc}}^{}}_{j}={{s_{}}_{\mathrm{sense}}^{}}_{i}|T_{j}|,$$

and the data of amount ${{s_{}}_{\mathrm{sense}}^{}}_{i}$ can be collected by the respective child node *j* and their descendant nodes.

The nodes that received ${{s_{}}_{\mathrm{alloc}}^{}}_{j}$ calculate the amount of data that the node themselves and their child nodes can collect in the above method and broadcast it to child nodes, and this process is iterated until ${s_{}}_{\mathrm{sense}}^{}$ of all leaf nodes is determined. Algorithm 2 shows the process of determining ${s_{}}_{\mathrm{alloc}}^{}$ and ${s_{}}_{\mathrm{sense}}^{}$. When ${s_{}}_{\mathrm{sense}}^{}$ is determined, the sensor node needs to use it to determine its own sensing period. If the period of the round is $p_{\mathrm{round}}$ and the amount of data sensed at one time is ${s_{}}_{\mathrm{unit}}^{}$, then data needs to be uniformly sensed for the number of times ${s_{}}_{\mathrm{sense}}^{}/{s_{}}_{\mathrm{unit}}^{}$ during the period of $p_{\mathrm{round}}$. The period of data sensing $p_{\mathrm{sense}}$ for collecting data of amount ${s_{}}_{\mathrm{sense}}^{}$ during one round is expressed as follows:

(25) $$p_{\mathrm{sense}}=p_{\mathrm{round}}\frac{{s_{}}_{\mathrm{unit}}^{}}{{s_{}}_{\mathrm{sense}}^{}}.$$

Sensor nodes collect the amount of data within their data transmission limits by sensing at every $p_{\mathrm{sense}}$.
**Algorithm 2:** Broadcasting the amount of data allocated to all child nodes.1:**if** Node *i* is a root node. **then**2:    ${{s_{}}_{\mathrm{alloc}}^{}}_{i}\leftarrow {{s_{}}_{\mathrm{aggr}}^{}}_{i}$3:**else**4:    Receiving ${{s_{}}_{\mathrm{alloc}}^{}}_{i}$ from its parent.5:**end if**6:${{s_{}}_{\mathrm{sense}}^{}}_{i}\leftarrow {{s_{}}_{\mathrm{alloc}}^{}}_{i}/|T_{i}|$7:${S_{\mathrm{alloc}}^{}}_{i}\leftarrow \emptyset$8:${S_{\mathrm{sense}}^{}}_{i}\leftarrow \left\{{{s_{}}_{\mathrm{sense}}^{}}_{j}|{{s_{}}_{\mathrm{alloc}}^{}}_{j}={{s_{}}_{\mathrm{aggr}}^{}}_{j}/|T_{j}|,j\in C_{i},{{s_{}}_{\mathrm{aggr}}^{}}_{j}\in {S_{\mathrm{aggr}}^{}}_{i},|T_{j}|\in {N_{\mathrm{sub}}^{}}_{i}\right\}$9:$k=|T_{i}|$10:Sorting ${S_{\mathrm{sense}}^{}}_{i}$ in ascending order.11:**for all**${{s_{}}_{\mathrm{sense}}^{}}_{j}\in {S_{\mathrm{sense}}^{}}_{i}$**do**12:      **if** ${{s_{}}_{\mathrm{sense}}^{}}_{j}>{{s_{}}_{\mathrm{sense}}^{}}_{i}$ **then**13:           ${{s_{}}_{\mathrm{alloc}}^{}}_{j}\leftarrow {{s_{}}_{\mathrm{sense}}^{}}_{i}|T_{j}|$14:      **else**15:           ${{s_{}}_{\mathrm{alloc}}^{}}_{j}\leftarrow {{s_{}}_{\mathrm{aggr}}^{}}_{j}$16:      **end if**17:      ${S_{\mathrm{alloc}}^{}}_{i}\leftarrow {S_{\mathrm{alloc}}^{}}_{i}\cup \left\{{{s_{}}_{\mathrm{alloc}}^{}}_{j}\right\}$18:      ${{s_{}}_{\mathrm{alloc}}^{}}_{i}\leftarrow {{s_{}}_{\mathrm{alloc}}^{}}_{i}-{{s_{}}_{\mathrm{alloc}}^{}}_{j}$19:      $k\leftarrow k-|T_{j}|$20:      ${{s_{}}_{\mathrm{sense}}^{}}_{i}\leftarrow {{s_{}}_{\mathrm{alloc}}^{}}_{i}/k$21:**end for**22:Broadcasting ${S_{\mathrm{alloc}}^{}}_{i}$ to all child nodes.

##### Example of Data Allocation Process

**Figure 3:**
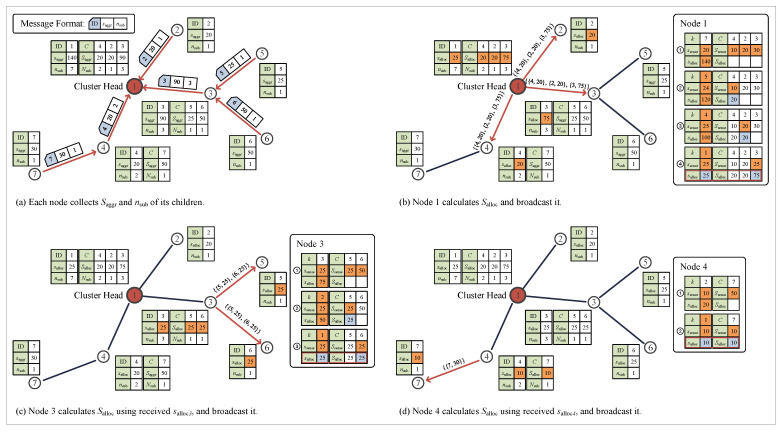
Data allocation process.

We now present an example to help understand the process of allocating data to be sensed at the nodes as described in the above section. Figure 3a–d illustrates the sequence of process in this example.

(**a**) After the process of routing, ${s_{}}_{\mathrm{aggr}}^{}$ is calculated for all individual nodes using Equation (12). Then, the leaf nodes (nodes 2, 5, 6, 7) send the information of ${s_{}}_{\mathrm{aggr}}^{}$ and $|T_{}|$ to parent nodes (nodes 1, 3, 4). When the parent nodes receive this information from all child nodes, the information is recorded, $|T_{}|$ is updated for the parent nodes, and the updated $|T_{}|$ and ${s_{}}_{\mathrm{aggr}}^{}$ are sent to the parent node (node 1). This process is iterated until the root node (node 1) receives all information of all child nodes. In this manner, the root node obtains the information regarding $|T_{1}|$, the number of all nodes in the MDT where the root node is a part of, and can obtain ${N_{\mathrm{sub}}^{}}_{1}=\left\{|T_{2}|,|T_{3}|,|T_{4}|\right\}$ and ${S_{\mathrm{aggr}}^{}}_{1}=\left\{{{s_{}}_{\mathrm{aggr}}^{}}_{2},{{s_{}}_{\mathrm{aggr}}^{}}_{3},{{s_{}}_{\mathrm{aggr}}^{}}_{4}\right\}$.

(**b**) According to Algorithm 2, ${{s_{}}_{\mathrm{alloc}}^{}}_{1}$ and ${S_{\mathrm{alloc}}^{}}_{1}$ are calculated for the root node (node 1) as shown on the right side of Figure 3b, and this information is broadcast to the child nodes. In this example, the total number of data that can be transmitted at the root node is 140, and there are 7 nodes in total for data transmission; the number of data that can be transmitted for each node is 20. Meanwhile, in the case of node 4, the total number of data that can be transmitted for two nodes is 20, and thus, the number of data that can be transmitted for each node is only 10. On the contrary, for node 3, the total number of data that can be transmitted for three nodes is 90, and thus, the number of data that can be transmitted for each node is 30. However, as a result of calculation at the 4th step in the figure, since the number of data that can be transmitted for each node for node 1 is only 25, in the case of node 3, the number of data that can be transmitted per node is also 25, leading to the total number of data allocated at 75 for transmission.

(**c**) Node 3, which has been allocated a total of 75 data for transmission from the root node, must share the allocated number of data with nodes 5 and 6. For this purpose, ${{s_{}}_{\mathrm{alloc}}^{}}_{3}$ and ${S_{\mathrm{alloc}}^{}}_{3}$ are calculated as shown on the right side of Figure 3c. As in the case of node 3, the total number of data that can be transmitted is 50 for node 6; however, since the allocated number of data for transmission per node is 25 for node 3, the number of data transmission at node 6 cannot exceed 25. Thus, the allocated number of data for transmission per node is also limited to 25 for node 6.

(**d**) In the case of nodes 4 and 7, as in the case of nodes 3 and 6, according to the allocated number of data for transmission at node 4, the allocated number of data for transmission is also limited to 10 for node 7.

As a result, all nodes are allocated ${s_{}}_{\mathrm{alloc}}^{}$, and ${s_{}}_{\mathrm{sense}}^{}$ is determined based on ${s_{}}_{\mathrm{alloc}}^{}$. By limiting data transmission exceeding the allocated amount of data, it is possible to prevent energy depletion of nodes in the hotspot such as root nodes, and ${s_{}}_{\mathrm{sense}}^{}$ is determined based on the principle of balanced distribution for all nodes.

In the proposed scheme as described so far, the MDT was composed considering the predicted available energy and energy consumption of the sensor nodes and the UAV, and the data that can be transmitted was obtained for each node. Based on the amount of data that can be transmitted for descendant nodes and the number of descendant nodes, the allocated amount of data that needs to be sensed by the root node and the descendant nodes was determined and broadcast to ensure balanced collection of data across the network. The nodes that received this information sensed data within the determined amount of data allocated and transmitted the sensed data accordingly. This approach ensures the prevention of energy depletion of nodes in the hotspot such as root nodes, while ensuring balanced data acquisition across all nodes involved.

## 4. Performance Evaluation

### 4.1. Simulation Environment

The performance of the proposed scheme was evaluated using simulations. For this purpose, SolarCastalia [47], a simulator was modified for application of a mobile sink node and WPT for use in this study. The proposed scheme in this study was compared with the following: (1) the existing method [48] modified for application of the energy-harvesting nodes (“Fixed”); (2) the existing method [48] with random selection of root nodes (“Random”); (3) a clustering method proposed by Yi and Yoon [44] (“Yi”); and (4) the method of adaptive data collection [27] proposed by us in our previous study (“Adaptive”).

In the Fixed method, a set number of root nodes of the MDT are placed in a predetermined location. In addition, root nodes are arranged at regular intervals to lower the depth of the MDT and transmission energy. In the Random method, as in the case of the Fixed method, root nodes are deployed at regular intervals, but the starting position is selected at random. In addition, to reduce the energy imbalance, a new node is chosen as the root node for every round. Yi’s method is similar to the Random method, but the number of MDTs is adjusted considering the energy of the UAV and sensor nodes and the amount of harvested energy. The Adaptive method proposed in our previous work determines the number of clusters by considering the energy of the UAV and the sensor nodes, and determines the amount of data to be collected by estimating the number of cluster members. Table 1 presents a summary of the main parameters used in the simulation. Common to all of these methods, the UAV travels a predetermined path and the root nodes are selected among nodes on the path of the UAV. When the UAV arrives at the root node, the node is wirelessly recharged and data aggregated in the root node is delivered to the UAV. The simulation was conducted for approximately 10 days, and the average value of results obtained from 50 repeated experiments was used. Table 1 represents the main parameters used in the simulation.

### 4.2. Simulation Results

#### 4.2.1. Performance Evaluation over Time

**Figure 4:**
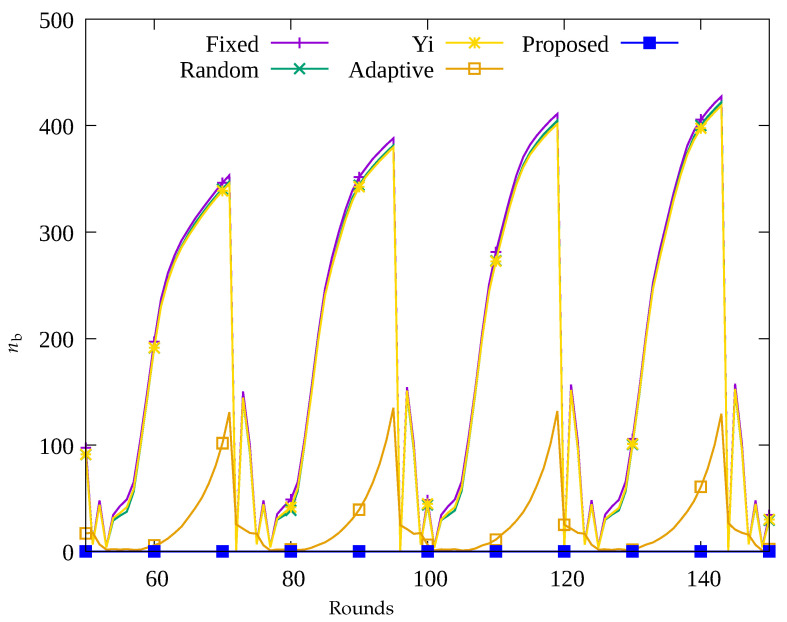
Change in the number of blackout nodes over time.

**Figure 5:**
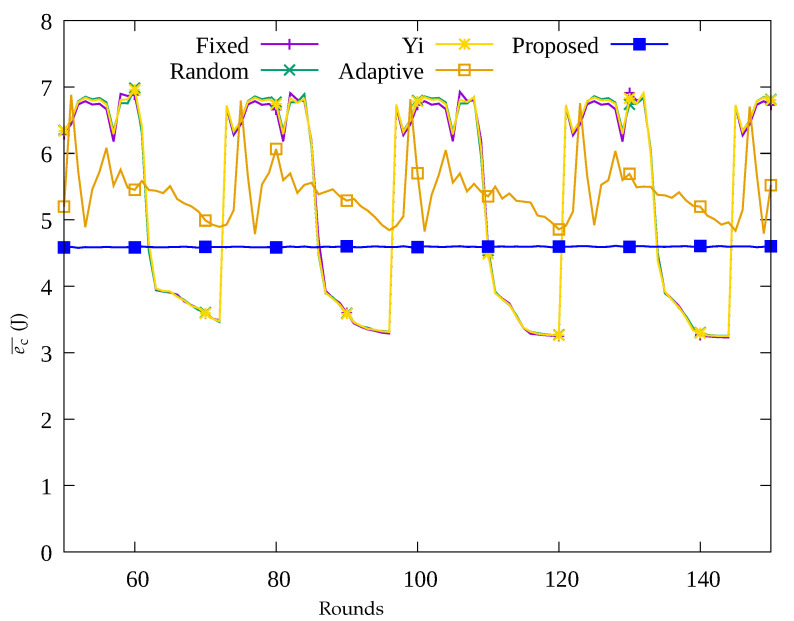
Change in the average amount of consumed energy over time.

**Figure 6:**
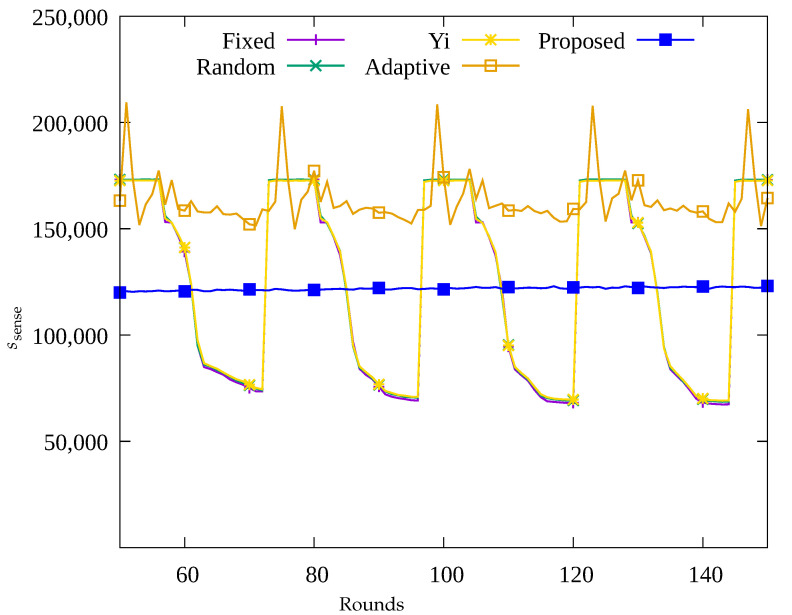
Comparison of the amount of data sensed over time.

**Figure 7:**
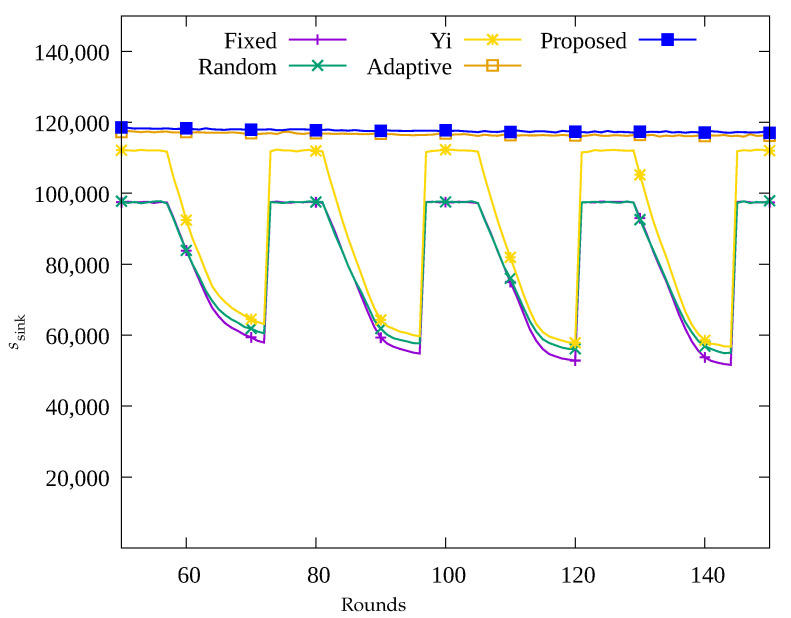
Comparison of the amount of data gathered at the sink node over time.

Figure 4, Figure 5, Figure 6 and Figure 7 show the changes in the following key variables over time, respectively: the number of blackout nodes (${n^{}}_{b}$), the average amount of energy consumed (${\bar{{e_{}}_{c}}}^{}$), the amount of data sensed by the sensors (${s_{}}_{\mathrm{sense}}^{}$), and the amount of data gathered by the UAV (${s_{}}_{\mathrm{sink}}^{}$). In Figure 4, since the sensor nodes use solar energy, it can be seen that ${n^{}}_{b}$ changes according to the change in solar energy over time in the Fixed, Random, and Yi methods. That is, when solar energy decreases at nighttime, many blackout nodes occur. Similarly, as shown in Figure 5, it can be seen that blackout nodes occur during the nighttime as a large amount of energy is consumed during the daytime. As a result, as shown in Figure 6, it can be seen that the number of data sensed by sensor nodes is large during the daytime and this number decrease during nighttime. Figure 7 indicates that although a significant amount of data was sensed during the daytime, a considerable portion of the data was lost while being transmitted to the sink node. This is because the nodes in the hotspot or those close to the root node, could not perform proper data relay, resulting in data loss during transmission. In Figure 4, it can be seen that since the Adaptive method uses an energy allocation scheme, the change in ${n^{}}_{b}$ according to the change in solar energy is small, but some blackout nodes occur due to the limitation of predicting the amount of data transmission. In addition, as shown in Figure 6, although a large number of data was sensed regardless of time by applying the energy allocation scheme in this method, 25% of the data is lost during transmission in Figure 7. This is thought to be due to the inaccuracy in prediction of amount of data to be collected, which results in poor performance in terms of data relay of the sensor nodes in hotspots. On the contrary, in the proposed scheme, as shown in Figure 4, almost no occurrence of blackout nodes was confirmed, and by limiting the amount of data collection, only the necessary amount of data is sensed and transmitted. As a result, the stable performance of data collection with UAV is provided by transmitting almost 100% of the sensed data as shown in Figure 6 and Figure 7, unlike the other methods used for comparison.

#### 4.2.2. Performance Evaluation According to Number of MDTs

**Figure 8:**
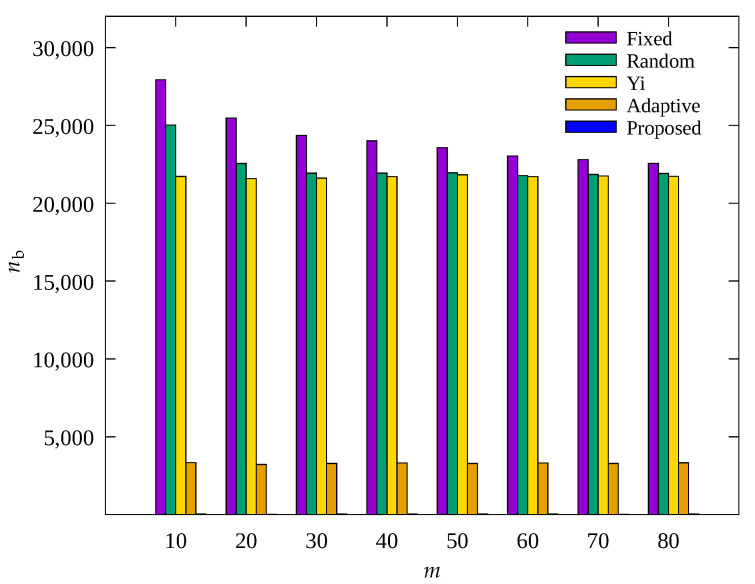
Change in the total number of blackout nodes according to number of MDTs.

**Figure 9:**
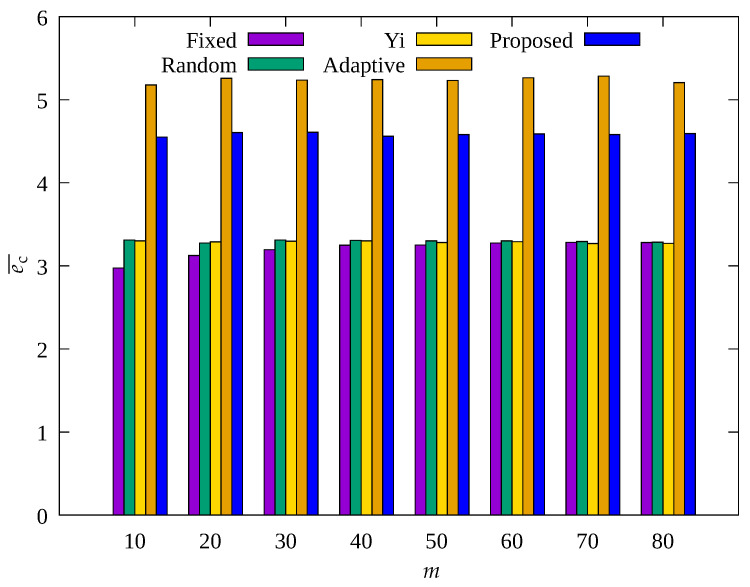
Comparison of the average amount of consumed energy according to number of MDTs.

**Figure 10:**
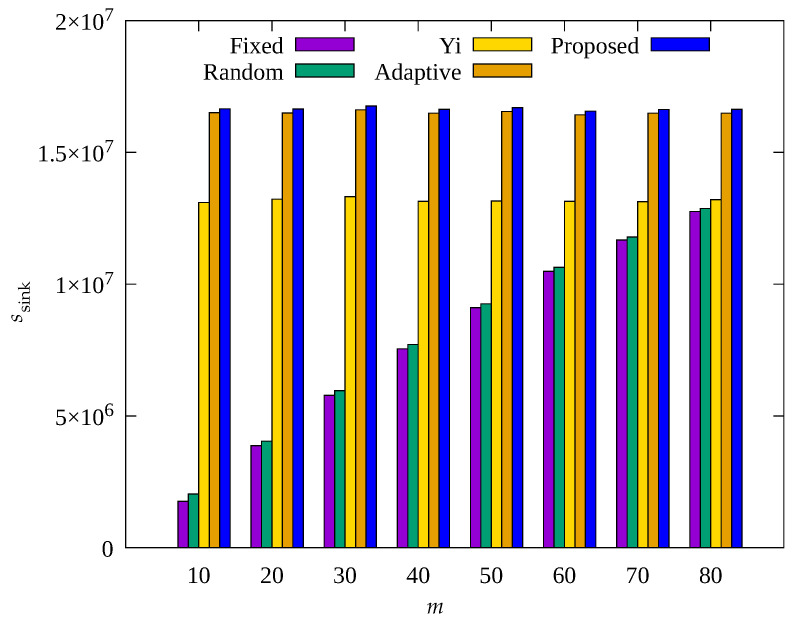
Comparison of the number of data gathered according to number of MDTs.

Figure 8, Figure 9 and Figure 10 show the changes in the following key variables according to the number of root nodes, $m_{}$, respectively: cumulative ${n^{}}_{b}$, ${\bar{{e_{}}_{c}}}^{}$, and, cumulative ${s_{}}_{\mathrm{sink}}^{}$. Yi and Adaptive schemes as well as the proposed scheme are based on adaptive control of the number of root nodes, indicating that the results of these schemes do not show change according to root nodes. However, since Fixed and Random methods are based on a fixed $m_{}$, it can be seen that the results vary depending on the set value of $m_{}$.

In Figure 10, it can be seen that ${s_{}}_{\mathrm{sink}}^{}$ shows a significant change in the Fixed and Random methods with increasing $m_{}$. This is because as $m_{}$ increases, the number of data transmission hops decreases, indicating a decrease in the amount of data to be transmitted by a single relay node. On the contrary, in Figure 8 and Figure 9, for Fixed and Random methods, with increasing $m_{}$, ${n^{}}_{b}$ shows a slight decrease and the average energy consumption shows a slight increase, but their results show no significant change overall. This is because as $m_{}$ decreases, data traffic is concentrated on a small number of nodes in the hotspot, and these nodes become blackout nodes, and the other nodes no longer have nodes for relaying their data, resulting in no data transmission and no energy consumption. It can be seen that Yi, Adaptive, and the proposed scheme, which are based on adaptive control of $m_{}$, show considerably more data collection than the other two schemes, and Adaptive and the proposed scheme show fewer blackout nodes than the other three schemes. From the above results, it can be seen that Yi, Adaptive, and the proposed scheme are capable of effective control of $m_{}$, and in particular, Adaptive and the proposed scheme properly adjust the amount of data sensed, resulting in a higher amount of data collection compared to other methods. Although the difference by less than 3% in ${s_{}}_{\mathrm{sink}}^{}$ for the proposed scheme is not remarkable compared to the Adaptive scheme, as shown in Figure 9, it can be seen that the proposed scheme shows about 10% less energy consumption than the nodes of the Adaptive scheme. This is because the proposed scheme controls the amount of data sensed by sensor nodes within limits, resulting in fewer unnecessary operations compared to the Adaptive scheme. In the proposed scheme, sensor nodes can transmit data more stably by using this extra energy for compression of forward error correction [49].

#### 4.2.3. Performance Evaluation According to Number of Sensor Nodes

**Figure 11:**
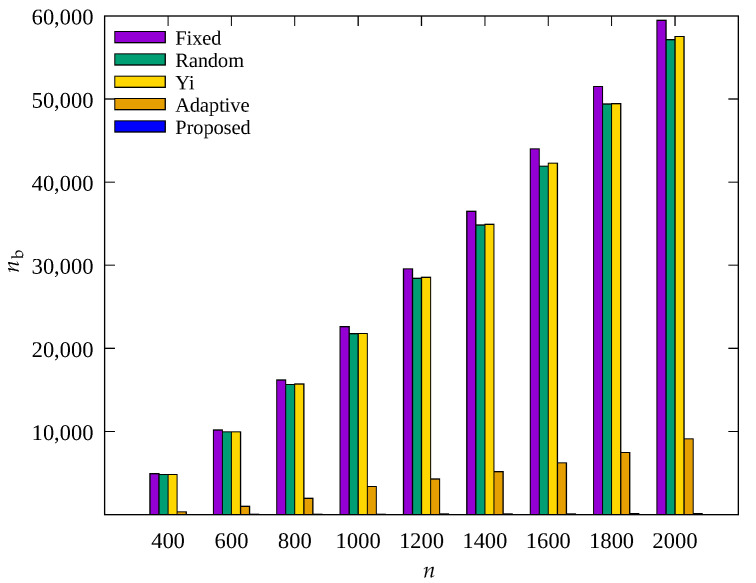
Change in total number of blackout nodes according to the number of nodes.

**Figure 12:**
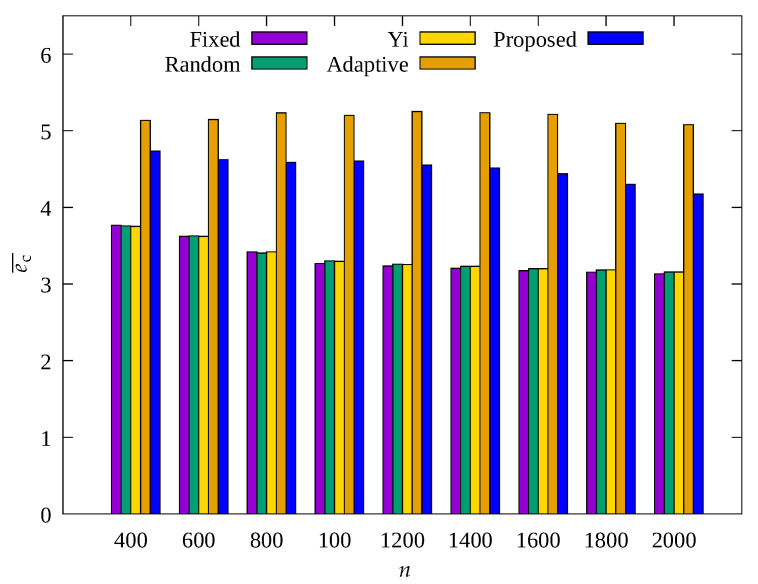
Comparison of the average amount of consumed energy according to the number of nodes.

**Figure 13:**
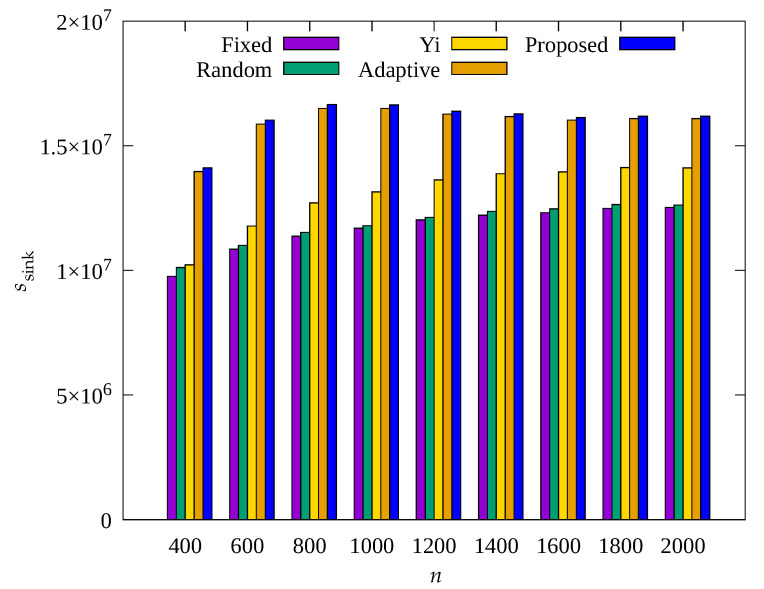
Comparison of the number of data gathered according to the number of nodes.

Figure 11, Figure 12 and Figure 13 show the changes in the following key variables according to the total number of sensor nodes, respectively: cumulative ${n^{}}_{b}$, ${\bar{{e_{}}_{c}}}^{}$, and cumulative ${s_{}}_{\mathrm{sink}}^{}$.

In Figure 11, it can be seen that, on the whole, ${n^{}}_{b}$ also increases as $n^{}$ increases. This is because with increasing $n^{}$, the number of transmission hops increases and the amount of data to be relayed by one root node increases, resulting in more energy consumption, which indicates poor scalability. The Adaptive scheme shows much smaller ${n^{}}_{b}$ than the Fixed, Random, and Yi techniques, but with increasing $n^{}$, ${n^{}}_{b}$ shows a gradual increase. However, it can be seen that the result obtained by the proposed scheme always shows almost no blackout nodes regardless of $n^{}$. This indicates that the occurrence of blackout nodes is effectively reduced by adaptive control of the amount of data collection according to the number of transmission hops regardless of $n^{}$ in the proposed scheme. Therefore, as shown in Figure 13, it can be seen that the proposed scheme and the Adaptive scheme show a greater ${s_{}}_{\mathrm{sink}}^{}$ than other schemes.

In Figure 12, it can be seen that the sensor nodes of the Adaptive technique show almost constant ${\bar{{e_{}}_{c}}}^{}$ regardless of $n^{}$, and the proposed scheme shows a decrease in ${\bar{{e_{}}_{c}}}^{}$ as $n^{}$ increases. It can be seen that the proposed scheme shows less energy consumption by controlling the amount of sensed data with limits to transmit less data to prevent blackout of the sensor nodes in the hotspot. As a result, it can be seen that the proposed scheme shows higher energy efficiency than the Adaptive scheme, but about 1% greater ${s_{}}_{\mathrm{sink}}^{}$, as shown in Figure 13.

#### 4.2.4. Performance Evaluation According to Node Density

**Figure 14:**
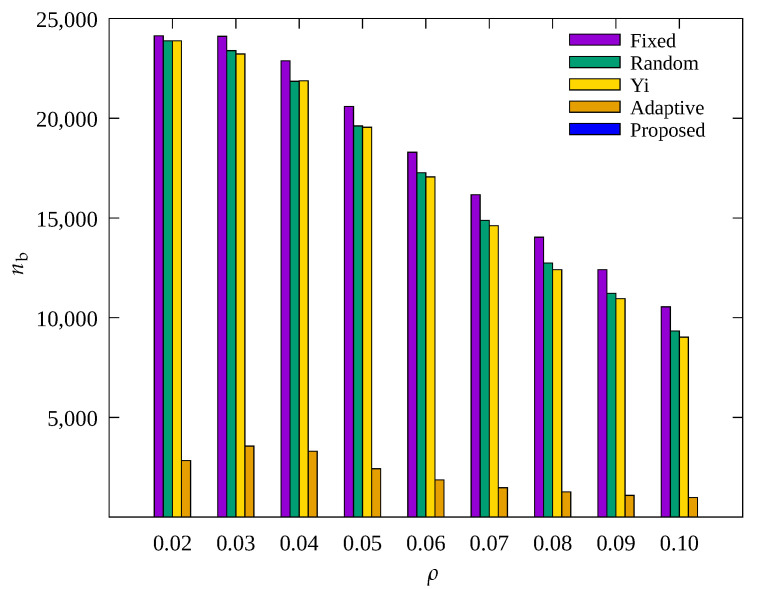
Change in total number of blackout nodes according to density.

**Figure 15:**
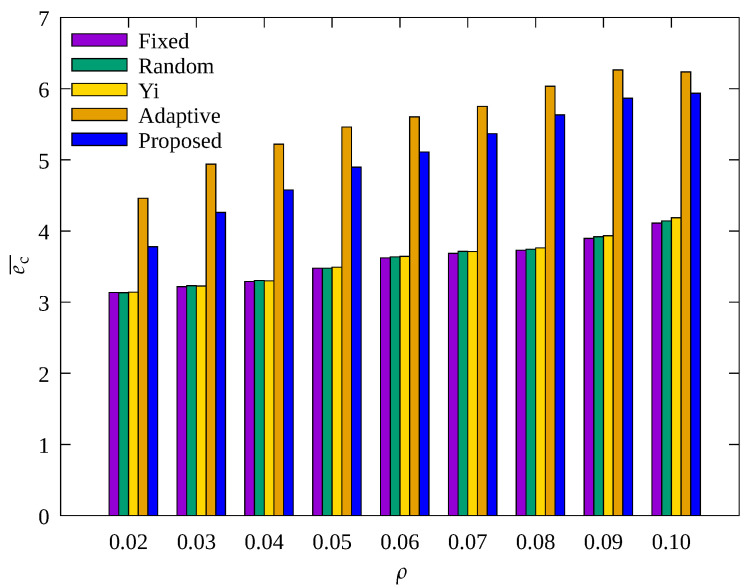
Comparison of the average amount of consumed energy according to density.

**Figure 16:**
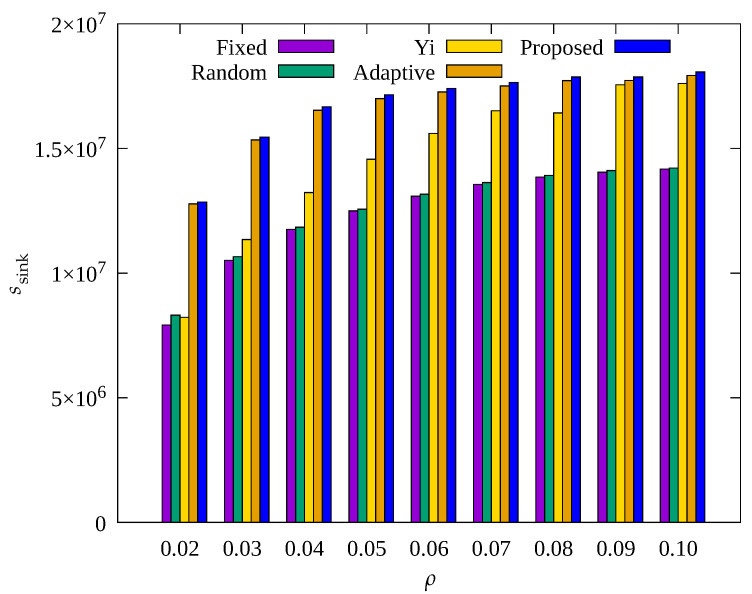
Comparison of the number of data gathered according to density.

Figure 14, Figure 15 and Figure 16 show the changes in the following key variables according to ρ, respectively: cumulative ${n^{}}_{b}$, ${\bar{{e_{}}_{c}}}^{}$, and cumulative ${s_{}}_{\mathrm{sink}}^{}$.

In Figure 14, it can be seen that ${n^{}}_{b}$ decreases as ρ increases in other schemes except for the proposed scheme. As ρ increases, the number of data transmission hops decreases, resulting in a decrease in the amount of data that relay nodes need to transmit. Therefore, the overall energy required for data transmission is reduced, and the occurrence of blackout nodes is reduced. However, the proposed scheme shows a stable performance in reducing blackout node occurrence. This indicates the proposed scheme effectively controls the amount of sensed data regardless of ρ. On the contrary, it can be seen in Figure 15 that ${\bar{{e_{}}_{c}}}^{}$ increases with increasing ρ. This is because with a decrease in blackout of the relay nodes, the data transmission paths of other nodes become effective, resulting in normal operation of data transmission by these other nodes, which leads to more energy consumption.

In Figure 16, it can be seen that as ρ decreases, ${s_{}}_{\mathrm{sink}}^{}$ shows a considerable decrease in all techniques, especially in the Yi technique. This is because, as discussed above, when ρ decreases, the number of transmission hops of all nodes increase, resulting in an increase in the load on the relay nodes. While ρ decreased from 0.1 to 0.02, ${s_{}}_{\mathrm{sink}}^{}$ in the Yi scheme decreased by 56%, whereas Adaptive and the proposed scheme only decreased by about 30%. This may be because these two techniques allow adaptive control of the amount of sensed data, and in particular, the proposed scheme shows about 3% more data gathered while consuming about 10% less energy as compared to the Adaptive technique, as in Figure 15. This indicates that the proposed scheme is superior in terms of the rate of data collection per unit energy consumed regardless of ρ.

As discussed above, we compared the amount of data gathered by the UAV and the number of blackout nodes according to time, the number of MDTs, the total number of nodes, and the node density to evaluate the performance of the proposed scheme. The simulation results showed that the proposed scheme is capable of adaptive control according to changes in many different factors, resulting in minimal occurrence of blackout nodes, minimal energy consumption, and acquisition of a larger amount of data.

The reason for the superior performance of the proposed scheme is that it determines the effective number of MDTs by considering the energy of the sensor nodes and the UAV, obtains accurate number of descendant nodes, and limits the amount of data transmission of the descendant nodes so as not to exceed the range of available energy for each node, thereby achieving accurate control of the amount of sensing data by nodes. As a result, the proposed scheme is capable of effectively reducing the occurrence of blackout for all nodes, especially nodes in the hotspot, thereby increasing the network availability and allowing collection of a larger amount of data regardless of the size of the network or node density. Furthermore, compared to other state-of-the-art techniques, for cases involving collection of similar amounts of data, the proposed scheme consumed about 10% less amount of energy on average, thereby increasing the available energy of the nodes. It is expected that the surplus energy will be utilized for data compression, in-network processing, or forward error correction, thereby increasing the energy efficiency of the sensor nodes and decreasing the packet error rate.

However, in the proposed scheme, the parent nodes strongly limits the amount of data sensed by the child nodes, to prevent the nodes go blackout as shown in Figure 8, Figure 11 and Figure 14. Correspondingly, due to excessive limiting, the energy of the leaf node may not be charged over the limit of the battery and may disappear. This energy imbalance cannot be easily resolved because the target environment of this scheme is the WSN in which an UAV repeatedly traverse the predefined path. This disadvantage will be improved in the future by changing the clustering or MDT formation method to disperse the hotspots.

## 5. Conclusions

This paper proposed a scheme, in the network environment of WRSNs with energy-harvesting sensor nodes, for applications in which a UAV is used for data gathering as well as for WPT-enabled recharging for nodes at the site of the visit. The proposed scheme employs adaptive control of data acquisition to attain enhanced energy efficiency while ensuring balanced data collection across all nodes. In the scheme, the data transmission path is determined by composing an efficient MDT, which factors in the surplus energy of the UAV, the allocated energy for each sensor node by time, and the number of descendant nodes. Then, by collecting the information on the amount of data that can be transmitted for each node and the number of descendant nodes, the amount of data that can be allocated for transmission for the root node and child nodes is determined and broadcast; in this manner, the amount of data relayed is limited, thus preventing energy depletion in the nodes located in hotspots. As a result, the occurrence of blackout nodes can be effectively prevented, while simultaneously ensuring balanced collection of a large amount of data. Upon comparative analysis of simulation results, it was confirmed that the proposed scheme, which employs adaptive control of data acquisition, achieves collection of a larger amount of data with less energy. In particular, the proposed scheme prevents indiscriminate sensing of unnecessary amount of data, thereby reducing energy consumption of the sensor nodes and load on relay nodes, which leads to minimal occurrence of blackout nodes. This scheme will be applied to applications that collect environmental data outdoors, such as climate measurement, to collect data uniformly and increase the lifespan of the network, thereby reducing network maintenance costs while collecting data effectively. In the future, further research needs to be performed on methods of increasing efficiency in energy utilization by making use of the surplus energy secured by preventing excessive sensing for data compression or in-network processing.

## Figures and Tables

**Table 1:** Simulation parameters.

Parameters	Values
$n^{}$	1000
ρ	0.04
Routing	MDT
Packet error rate	5%
Duration of a round	1 h
Transmission period	1 min
WPT efficiency	50%
Transmission range	10 m
Transmission rate	250 kbps
Sensor battery capacity	110 mAh
Sensor initial energy	55 mAh
α	4
β	100 pJ/bit/$m^{\alpha }$
${e_{}}_{\mathrm{Rx}}^{}$	48 mJ
${e_{}}_{\mathrm{idle}}^{}$	8 μJ
Max UAV speed	16 m/s
Max UAV flying time	20 min
UAV Battery capacity	4480 mAh

## Data Availability

The data presented in this study are available on request from the corresponding authors.

## Abbreviations

The following abbreviations are used in this manuscript:

WSN	Wireless sensor network
WRSN	Wireless rechargeable sensor network
UAV	Unmanned aerial vehicle
WPT	Wireless power transfer
MDT	Minimum depth tree
RF	Radio frequency
